# A Control-Theoretical System for Modulating Hippocampal Gamma Oscillations Using Stimulation of the Posterior Cingulate Cortex

**DOI:** 10.1109/TNSRE.2022.3192170

**Published:** 2022-08-17

**Authors:** Carlos E. Davila, David X. Wang, Maxwell Ritzer, Rosalyn Moran, Bradley C. Lega

**Affiliations:** Department of Electrical and Computer Engineering, Southern Methodist University, Dallas, TX 75205 USA; Department of Electrical and Computer Engineering, Southern Methodist University, Dallas, TX 75205 USA; Department of Neurological Surgery, University of Texas Southwestern Medical Center, Dallas, TX 75390 USA; Department of Neurological Surgery, University of Texas Southwestern Medical Center, Dallas, TX 75390 USA; Department of Neuroimaging, King’s College London, Institute of Psychiatry, Psychology, and Neuroscience, London WC2R 2LS, U.K.; Department of Neurological Surgery, University of Texas Southwestern Medical Center, Dallas, TX 75390 USA

**Keywords:** Hippocampus, gamma oscillations, closed-loop modulation, open-loop stimulation, posterior cingulate cortex

## Abstract

Closed-loop stimulation for targeted modulation of brain signals has emerged as a promising strategy for episodic memory restoration. In parallel, closed-loop neuromodulation strategies have been applied to treat brain conditions including drug-resistant depression, Parkinson’s Disease, and epilepsy. In this study, we seek to apply control theoretical principles to achieve closed loop modulation of hippocampal oscillatory activity. We focus on hippocampal gamma power, a signal with an established association for episodic memory processing, which may be a promising ‘biomarker’ for the modulation of memory performance. To develop a closed-loop stimulation paradigm that effectively modulates hippocampal gamma power, we use a novel data-set in which open-loop stimulation was applied to the posterior cingulate cortex and hippocampal gamma power was recorded during the encoding of episodic memories. The dataset was used to design and evaluate a linear quadratic integral (LQI) servo-controller in order to determine its viability for in-vivo use. In our simulation framework, we demonstrate that applying an LQI servo controller based on an autoregressive with exogenous input (ARX) plant model achieves effective control of hippocampal gamma power in 15 out of 17 experimental subjects. We demonstrate that we are able to modulate gamma power using stimulation thresholds that are physiologically safe and on time scales that are reasonable for application in a clinical system. We outline further experimentation to test our proposed system and compare our findings to emerging closed-loop neuromodulation strategies.

## I. Introduction

The last ten years have witnessed a flourishing in the development of therapeutic brain stimulation to treat memory disorders. Nascent therapies for memory benefit from antecedent experience in neuromodulation targeting Parkinson’s disease [1], [2], epilepsy [3], and depression [4]–[6], mostly in the form of open loop stimulation. Open-loop stimulation relies on manually setting stimulation parameters for each patient via a trial-and-error procedure that is guided by clinical assessment of symptoms [1]. Such open-loop stimulation fails to account for the fast dynamics of electrophysiological signal during cognition, although it has proven effective for movement disorders. When applied for the neuromodulation of memory (unlike movement disorders), open-loop strategies have not only largely failed to demonstrate benefit in memory performance, but have been shown to worsen memory [7]–[11]. Stimulation paradigms that have shown greater promise in improving memory rely on responsive, closed-loop stimulation, in which a neural feedback signal guides subsequent stimulation pulses [12]–[14]. Closed loop neuromodulation strategies emerged from efforts within the DARPA Restoring Active Memory (RAM) program [15]. The effort to which we contributed, which reported a 15% average increase in memory performance across 40 participants, used logistic regression-based classifiers to predict encoding success [12]. Classifiers were trained for each patient from approximately 700 milliseconds of brain recordings over 90 or more intracranial electrodes. The trained classifier decoded the likelihood of successful encoding following item presentation within an episodic memory task and delivered during ‘unfavorable’ brain states with a low likelihood of encoding success. While promising, several concerns remain that may impede the practical implementation of such a device. The first is a limit in classifier performance and generalizability across subjects and experimental sessions, requiring bespoke models uniquely trained for each patient [16]. Further, this method requires a cumbersome empirical parameter identification routine to identify the appropriate brain region and stimulation characteristics needed to predictably alter brain activity. Finally, the logistic regression models used for prediction of encoding success (and control of stimulation) required extensive patient data from over 100 recording contacts, more than would be feasible in a clinically-applicable system. Results remain preliminary overall, but these reports highlight the potential of closed loop approaches for memory restoration.

Shanechi describes an alternative closed-loop, neuromodulation method in the context of treating depression [17]. The strategy incorporates an oscillatory signal acting as a ‘biomarker’ of mood, namely orbitofrontal theta or alpha power, which is used as a signal amenable to control using state space modeling and an LQI servo-controller. A proposed testbench for incorporating this approach was described by Yang et al (2018). Their proposed system implements a Kalman filter to estimate the biomarker signal and an LQR controller to manipulate it precisely [18]. Here, we seek to apply some of the principles described in this approach to neuromodulation strategies for memory disorders. Two essential questions must be considered when developing this approach: 1) what brain signal can serve as an effective ‘biomarker’ for memory, and 2) what stimulation strategy can modulate this biomarker safely and effectively? Hippocampal gamma oscillatory power is a logical choice to address the first question. Both animal and human studies have established that changes in gamma oscillatory power predict memory success, along with participating in local and regional coupling via phase synchrony and cross frequency coupling [19], [20]. Regarding a strategy for modulating hippocampal gamma oscillations, we recently published data demonstrating that stimulation of the posterior cingulate cortex (PCC) reliably elicits increases in hippocampal gamma band oscillatory power during episodic memory processing [8]. The PCC represents a promising target for neuromodulation given its dense connectivity to diverse brain regions, including participation in the default mode network [21], [22]. These data were collected using an open-loop stimulation paradigm (with overall reduction in memory performance, as with previous publications targeting brain locations other than the PCC). However, these data allowed us to model hippocampal gamma power in the presence and absence of stimulation during memory behavior, which facilitated the construction of a control model for the modulation of this signal.

Our efforts establish the feasibility of a control system for memory neuromodulation predicated on posterior cingulate stimulation using linear system identification methods similar to those reported previously [18]. We show that we are able to model the relationship between PCC stimulation and responsive hippocampal gamma power using our ARX framework, accurately representing the gamma power time series in both stimulation and non-stimulation conditions. Next, we show that a system using PCC stimulation to modulate hippocampal gamma power is controllable in 100% of subjects as measured by computing the rank of the controllability matrix [23]. We then describe a simulation framework for the PCC–hippocampal system constructed using Simulink.

## II. Materials and Methods

### A. Participants

A total of 18 participants (ages 20–60, 9 female) with medication-resistant epilepsy who underwent stereo-electroencephalography surgery with the goal of identifying their ictal onset region(s) participated in the study. Participants came from our epilepsy surgery program across a time of span of 4 years. Only patients who had intracranial electrodes placed within the posterior cingulate were included in the study. The research protocol was approved by the UT Southwestern Medical Center Institutional Review Board (082014–075 on 08/2014), and each participant gave informed consent prior to data collection. Following implantation, electrode localization was achieved by co-registration of the post-operative computer tomography scans with pre-operative magnetic resonance images using the FLIRT software package (https://fsl.fmrib.ox.ac.uk/fsl/fslwiki/FLIRT). The co-registered images were evaluated by a member of the neuroradiology team to determine the final electrode locations.

### B. Experimental Task

Each subject participated in a verbal free-recall task in which they studied a list of words with the intention to commit the items to memory. The task was performed at bedside on a laptop. In the encoding phase, lists of 10 words were visually presented. Words were selected at random from a pool of high frequency English nouns (http://memory.psych.uppen.edu/WordPools). Each word was presented for 1500 ms, followed by a blank inter-stimulus interval of 1000 ms. Stimulation to the PCC was applied during the entire duration of the encoding phase and was synchronized with the onset of the first word in each list. All items were encoded in the presence of stimulation. After presenting the word list, a post-encoding delay was followed for 20 seconds during which there was no stimulation. During this delay, each subject performs an arithmetic task to limit rehearsal. Math problems of the form A + B + C = ?? were given to each subject, with the values of A, B, and C set to random single digit integers. Subsequently, the recall period started (after 20 seconds of math) with an auditory tone of 60 Hz of a 300 millisecond duration. Subjects were instructed to recall as many words as possible from the previously presented word list within this 30 second recall period (memory retrieval). During this recall interval, there was no stimulation. Vocal responses were digitally recorded and parsed offline using Penn TotalRecall (http://memory.psych.upenn.edu/TotalRecall). Further details of the task are described previously in extensive published work [8], [19]. The experiment included stimulation and non-stimulation item lists. The non-stimulation lists were interleaved with stimulation lists in a pseudo random fashion. This allowed us to compare the effects of stimulation on gamma power and control for gamma power increases resulting from task-induced gamma.

### C. Electrical Stimulation

Stimulation was applied to the PCC with an amplitude of 2 mA and a frequency of 100 Hz using the Grass S88 stimulator (Grass Technologies). Stimulation parameters were determined using accepted safety thresholds for DBS drawn from initial work [24] and by incorporating typical parameters used for DBS techniques [9], [11]. We used bipolar pairs of electrodes for stimulation with the deepest contact localized to the PCC. The critical safety threshold that is generally accepted is 30 *μ*C*/*cm^2^/phase [25]. Here, we used biphasic matched-square wave pulses with a pulse width of 200 *μ*s. The surface area of depth electrodes is 0.05 cm^2^. All of the 18 participants had their stimulation sites in the left PCC.

For this study, we used the first word presented from each list. Since these words were preceeded by the 50-second post-encoding delay and recall tasks, during which there was no stimulation, the stimulus amplitude took the form of a step function going from zero to 2 mA at the onset of the presentation of the first word (encoding event). Each subject exhibited a total of 10 such encoding events. We chose to focus on these events since it allowed us to compare gamma power level over a 2 second period prior to stimulation onset with gamma power during a 2 second interval after the onset of stimulation. This eliminated the effects of changes in baseline gamma over longer periods of time that can result from nonstationarity in the iEEG.

### D. Electrocorticographic Recordings and Simulation Framework

Intracortical electroencephalogram (iEEG) signals were recorded via depth electrodes (contacts spaced 5–10 mm apart) using a Nihon-Kohden EEG system under a bipolar montage with the most medial white matter contact on individual electrodes as the reference (for hippocampal recordings, this was white matter in the adjacent subcortical temporal lobe). For each subject, ten 4-second trials corresponding to the first encoding event for each list were collected using a sampling interval of 0.002 seconds. Channels exhibiting highly nonphysiologic signals due to damage or misplacement were excluded prior to re-referencing. We utilized a bipolar referencing scheme to reduce the effect of stimulation artifact from affected electrodes modifying signal at other locations. A projection-based 60 Hz notch filter was applied. The notch filter has zero phase distortion and narrow notch width [26]. In addition, a linear phase FIR notch filter having a notch width of 98–102 Hz was applied to filter out stimulus artifact at 100 Hz. One subject who showed significant nonstationarity in baseline gamma power during the recording epochs was removed from the study, leaving a total of 17 subjects.

**Fig. 1.**
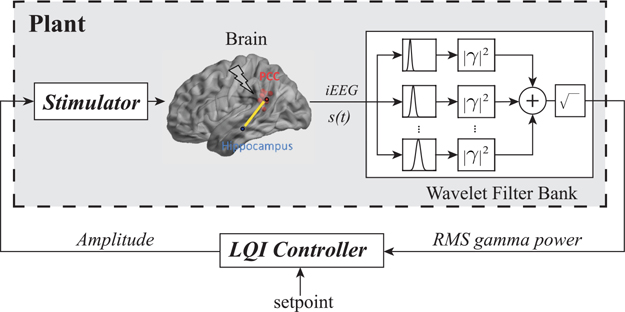
Simulation framework for control of RMS gamma power.

Our simulation framework consists of a plant model that generates gamma power and a closed-loop LQI controller that attempts to drive measured gamma power towards a designated setpoint. The plant consists of a stimulator, the brain, iEEG measurement instrumentation, and a wavelet filter bank that estimates instantaneous RMS gamma power (see Figure 1). The input to the plant is the stimulus amplitude, and the output is estimated RMS gamma power. Intracranial EEG (iEEG) is passed through an analytic wavelet filter bank consisting of Morse wavelets with symmetry parameter equal to 3 and time-bandwidth product equal to 60 [27]. We used 10 wavelet filters covering three gamma frequency sub-bands of 30–50 Hz, 50–70Hz, and 70–90 Hz. Estimates of instantaneous RMS gamma power were obtained by taking the square root of the sum of the squared magnitudes of the wavelet filter outputs, as shown in Figure 1. Analytic wavelet filters were found to have greater sensitivity in detecting short-duration gamma oscillations compared to using a bandpass filter followed by conversion to analytic signal via the Hilbert transform. In our simulation framework, the plant is modeled using an ARX model as described in the following section.

### E. Modeling Gamma Power in the Hippocampus

Rather than model iEEG, our approach seeks to directly model hippocampal gamma power. Modelling the full spectrum of iEEG would have necessitated arriving at a model that generates gamma power with the same statistical properties as our experimentally measured gamma power, and would have presented a more challenging modeling problem. The selection of a suitable model for hippocampal gamma power was based on the following criteria:

1. The model should exhibit random fluctuations in instantaneous gamma power regardless of whether the brain is being stimulated or not.
2. The power spectral density (PSD) of the model output should closely match that of actual RMS gamma power.
3. The model should predict experimentally measured mean RMS gamma power during stimulation as well as in the absence of stimulation.
4. Inputs and outputs for the model should reflect physiologically relevant quantities, namely limiting the amplitude of stimulation to less than 9 mA in order to insure that the current will be physiologically safe. [25].
5. The model should be as simple as possible in order to minimize the computational complexity of the controller.

We modeled gamma power using an autoregressive model with exogenous input (ARX) model, given by,

(1) $$x(t)=-\sum_{k=1}^{p}a_{k}x(t-k)+b_{DC}u_{DC}+b_{s}u_{s}(t)+w(t)$$

Here, *x*(*t*) represents the instantaneous RMS gamma power having units of *μ*V at discrete-time *t*. The exogenous input *u*_*DC*_ is a constant that determines the mean value of the RMS gamma power when the stimulus current *u*_*s*_(*t*) = 0. Both *u*_*DC*_ and *u*_*s*_(*t*) are currents having units of mA, corresponding to the amplitude of the stimulus current. This choice of units for the input is justified since the power in a periodic signal is proportional to its amplitude. For example for a sinusoidal signal *A*_*c*_ cos(*ω*_*c*_*t* + *θ*), the root mean square (RMS) power is given by $A_{c}/\sqrt{2}$, independently of the frequency *ω*_*c*_ and phase *θ*. From the Fourier series representation of periodic signals and Parseval’s theorem, the result holds for any periodic signal. Therefore setting *u*_*s*_(*t*) equal to the stimulus amplitude will proportionally affect the mean RMS gamma power. The quantity *w*(*t*) is a zero-mean Gaussian white noise process having variance $\sigma_{w}^{2}$ and units of *μ*V. This noise is not measurable and produces random fluctuations in the RMS gamma power. The ARX model parameters, *a*_*k*_*, k* = 1, . . ., *p* are dimensionless, while *b*_*DC*_ and *b*_*S*_ have units of resistance (m). All model parameters can be identified from experimental iEEG data for given values of *u*_*DC*_ and *u*_*s*_. We chose *u*_*DC*_ = 1 mA, this value is not critical since *u*_*DC*_ will be scaled by *b*_*DC*_ to provide the correct DC value for the gamma signal. An expression for the mean RMS gamma power predicted by the model can be established by ignoring the random signal *w*(*t*) and using the Final Value Theorem. The result is,

(2) $$\underset{t\to \infty }{\lim}x(t)=\frac{b_{DC}u_{DC}\;+\;b_{s}u_{s}}{1\;+\;\sum_{k=1}^{p}a_{k}}\equiv \hat{\gamma }$$

Equation (2) can be used to predict the mean RMS gamma power for any fixed amplitude stimulus current with amplitude *u*_*s*_. In our model, *u*_*s*_ was the step function

(3) $$u_{s}(t)=\{\begin{array}{ll} 0, & t=1,\ldots ,N/2\\ 2, & t=N/2+1,\ldots ,N \end{array}$$

where *N* is assumed to be even. Using (2), the mean gamma power for no-stimulation is

(4) $${\hat{\gamma }}_{ns}\equiv \frac{b_{DC}}{1\;+\;\sum_{k=1}^{p}a_{k}}$$

while for the stimulation case, it is given by

(5) $${\hat{\gamma }}_{s}\equiv \frac{b_{DC}\;+\;2b_{s}}{1\;+\;\sum_{k=1}^{p}a_{k}}$$

Using well known relationships between the power spectral density (PSD) of the input and output of a linear time-invariant system, we can arrive at an expression for the PSD of our ARX model:

(6) $$P_{xx}(f)=\frac{\sigma_{w}^{2}\;+\;4b_{s}^{2}/{\left(2\pi f/F_{s}\right)}^{2}}{{\left|1\;+\;\sum_{k=1}^{p}a_{k}e^{-j2\pi fk/F_{s}}\right|}^{2}}$$

where *f* is continuous-time frequency in Hz and *F*_*s*_ is the sampling frequency in Hz [28]. We will use this expression to compare the theoretical PSD of our model with the estimated PSD of experimentally measured instantaneous gamma power.

### F. Identification of ARX Model

The ARX model parameters can be estimated with a least squares linear prediction approach using only the ARX data samples *x*(*t*), and the exogenous inputs, *u*_*DC*_*, u*_*s*_(*t*)*, t* = 1, . . ., *N*. This approach seeks to minimize the quantity

(7) $$\epsilon_{pred}=\sum_{t=p+1}^{N}e{\left(t\right)}^{2}$$

over the ARX model parameters, *a*_*k*_*, k* = 1, . . ., *p, b*_*DC*_, and *b*_*s*_, where the prediction errors are given by

(8) $$e(t)=x(t)-\sum_{k=1}^{p}a_{k}x(t-k)-b_{DC}u_{DC}-b_{s}u_{s}(t)$$

with *t* = *p* + 1, . . ., *N*. In matrix notation, (8) represents an overdetermined system of equations,

(9) Cv≈d

with

(10) $$C=\left[\begin{array}{ccccc} x(p) & \cdots & x(1) & u_{DC} & u_{s}(p+1)\\ x(p+1) & \cdots & x(2) & u_{DC} & u_{s}(p+2)\\ \vdots & \ddots & \vdots & \vdots & \vdots \\ x(N-1) & \cdots & x(N-p) & u_{DC} & u_{s}(N-p) \end{array}\right]$$

*d* = [*x*(*p* + 1)*x*(*p* +·2)· · ·*x*(*N*)*]*^*T*^ and the ARX parameter vector *v = [a*_1_ *a*_2_· · · *a*_*p*_ *b*_*DC*_ *b*_*s*_]^*T*^. It is well known that these least squares equations have a unique solution provided that the matrix *C* has full column rank [29]. This is discussed in detail in Section VI. The least squares solution to (9) is given by $v^{*}={\left(C^{T}C\right)}^{-1}C^{T}d$, although in practice, it is more computationally efficient and accurate to compute a QR decomposition of *C* [30]. A commonly used quality metric for ARX models is the minimum mean-squared prediction error, which can be estimated using the squared norm of the minimum prediction error vector [31]

(11) $$\epsilon_{pred}^{*}=\frac{1}{N}{\left\|Cv^{*}-d\right\|}^{2}$$

where Cv* is the optimal least squares prediction of the values in *d* and ∥·∥ is the vector 2-norm. A normalized measure which takes into account the variance of *x*(*t*) is the “fit percentage”, given by

(12) $$\text{FitPerc}=100\left(1-\frac{\sqrt{\epsilon_{pred}^{*}}}{\left\|d\;-\;\mu_{d}\right\|}\right)$$

where *μ*_*d*_ is the sample mean of the data vector *d* [32]. Note that a low mean squared prediction error leads to a fit percentage close to 100%. In Section VI-C we demonstrate that the linear prediction approach to identifying the parameters of our ARX model gives good results.

### G. State-Space Model

Our controller requires that the model be in state-space form, given by

(13) $$x_{t+1}=Ax_{t}+Bu_{t}+Gw(t)\;y\left(t\right)=Cx_{t}+Du_{t}+v\left(t\right)$$

where *x*_*t*_ is the system state vector at discrete-time *t*, *u*_*t*_ = [*u*_*DC*_ *u*_*s*_]^*T*^ and *y*_*t*_ are scalar input and measurements, respectively, and *w*(*t*), and *v*(*t*) are the (scalar) system disturbance and observation noise signals, respectively. The dimensions of these vectors, as well as that of matrices *A, B, C, D,* and *G*, depend on the state-space model. The ARX model can be readily implemented using a state-space model. In our case, the state vector consists of *p* consecutive samples of the RMS gamma power signal, *x*_*t*_ = [*x*(*t*) *x*(*t* − 1) ··· *x*(*t* − *p* + 1)]^*T*^. Both the observation *y*(*t*) = *x*(*t*) and the system disturbance *w*(*t*) are scalar quantities. The observation noise *v*(*t*) accounts for measurement noise and modeling uncertainties. Correspondingly, in order for (13) to agree with (1), we must have:

(14) $$A=\left[\begin{array}{ccccc} -a_{1} & -a_{2} & \cdots & -a_{p-1} & -a_{p}\\ 1 & 0 & \cdots & 0 & 0\\ 0 & 1 & \cdots & 0 & 0\\ \vdots & \vdots & \ddots & \vdots & \vdots \\ 0 & 0 & \cdots & 1 & 0 \end{array}\right]B=\left[\begin{array}{cc} b_{DC} & b_{s}\\ 0 & 0\\ \vdots & \vdots \\ 0 & 0 \end{array}\right]$$

*G* = [1 0 ··· 0]^*T*^, *C* = [1 0 ··· 0], and *D* = 0. The choice of an ARX model has an important advantage compared to a general linear state-space model (LSSM). Since the state vector consists of consecutive samples of the RMS gamma power, there is no need to estimate the state vector using a Kalman filter.

### H. LQI Servo-Controller

**Fig. 2.**
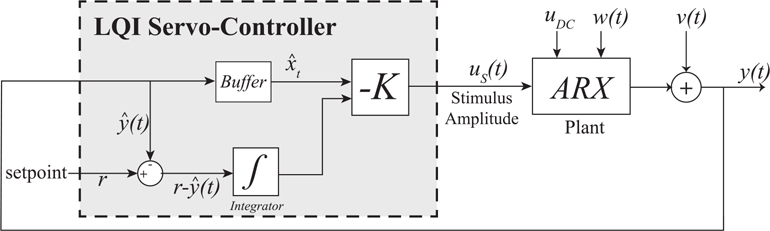
Linear quadratic integral servo-controller.

When stimulating to control gamma power, we are faced with two competing goals: to bring gamma power to some predetermined setpoint *r* as quickly as possible while minimizing the amount of stimulus energy delivered to the patient. These conflicting aims lend themselves to employing linear quadratic integral (LQI) control [33]. The cost function for our controller takes the form:

(15) $$J=\sum_{t=0}^{\infty }z_{t}^{T}Qz_{t}+Ru_{s}{\left(t\right)}^{2}$$

where $z_{t}={\left[x_{t}^{T}\;e_{i}(t)\right]}^{T}$, and

(16) $$e_{i}(t)=T_{s}\sum_{k=0}^{t}r-y(t)$$

is the discrete-time integration of the difference between the gamma power setpoint *r* and the observed gamma power *y*(*t*). The user sets the parameters *Q* and *R*, in order to tune the controller’s performance. The parameter *Q* adjusts the rate at which gamma power approaches the setpoint, while the parameter *R* determines the amount of stimulus energy delivered to the patient via the stimulus amplitude *u*_*s*_(*t*). This optimal control problem has a well-known solution, the cost function in (15) is minimized using the control law $u_{s}\left(t\right)=-K_{z_{t}}$, where *K* is the solution to an algebraic Ricatti equation that depends on the state-space model *A, B, C, D, G*. The controller takes the buffered plant output as the state vector *x*_*t*_, which after augmenting with the integrated setpoint error *e*_*i*_(*t*), is multiplied by the gain vector −*K* to determine the stimulation current amplitude. The resulting LQI servo-controller is shown in Fig. 2. In practice, the optimal set point can be modified based on subject-level empirical observations. We assigned ‘guardrails’ to the maximum stimulation amplitude delivered by the Blackrock device with a maximum of 9 mA to reflect safety requirements; this kept stimulation well-within the approximately 25 *μ*C per cm^2^ employed in clinical systems [25], [34].

## III. Results

### A. Prediction of Mean RMS Gamma Power Levels With ARX Models

**Fig. 3.**
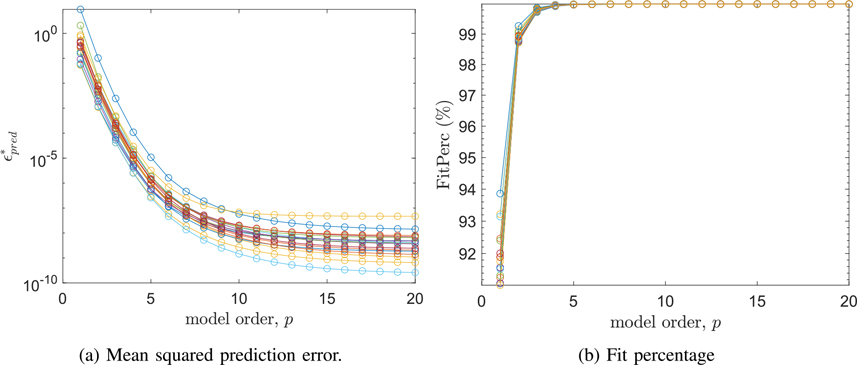
Model quality metrics for all subjects.

The Matlab “arx” function was then used to estimate ARX models for each of the 15 subjects who experienced RMS gamma power increases, based on the 10 trials where the stimulus was a step function as described in Section II-C. We used the exogenous inputs given by *u*_*DC*_ = 1 mA and *u*_*s*_(*t*) given by (3). The ARX models derived for each trial were then averaged to form a composite ARX model for each subject. The mean squared prediction error $\left(\epsilon_{pred}^{*}\right)$ and fit percentage (FitPerc) quality metrics were computed for *p* = 1, . . ., 20, and are shown in Fig. 3. To select a model order, we concluded that *p* = 6 offered a reasonable trade-off between mean-squared prediction error, fit percentage, and computational complexity. At *p* = 6 all but one subject had mean-squared prediction error values lower than 10^−6^. Moreover, fit percentage approached 100% for model orders satisfying *p* ≥ 4. In order to check that these results were not due to over-training, we performed 10-fold cross validation, by computing an ARX model based on 9 of 10 trials and checking the ability of the model to predict the data in the 10th trial, then averaging over all test trials. There was virtually no change in the model quality metrics. The mean 10-fold cross validation fit percentage was 99.9930% averaged over all subjects, compared to 99.9931% when using the model for the test trial.

**Fig. 4.**
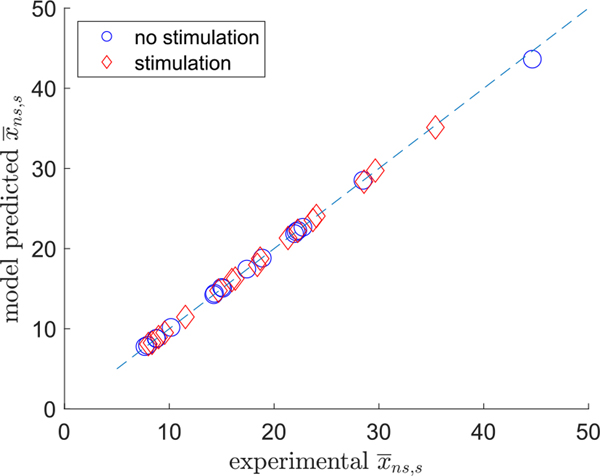
Mean RMS gamma power level predictions by ARX model.

Given the results of the previous section, we conclude that the ARX model is adequate for instantaneous RMS gamma power. Next we compared the model predictions of mean RMS gamma power for the no-stimulus and stimulus intervals with actual quantities. The results are shown in Fig. 4 and show good agreement between experimental and model-predicted mean RMS gamma power levels for both stimulation and non-stimulation conditions.

Another goal was that our model should closely match the power spectral density (PSD) of instantaneous RMS gamma power. For each subject, we computed the periodogram of each of the 10 trials (using a *N* = 2000-sample Hanning window) and averaged them. The averaged periodograms were then compared to the theoretical power spectral density for the identified ARX model (see (6)). Typical results for several subjects are shown in Figure 6. From around 0–100 Hz, there is a fairly close match between the periodogram estimate of instantaneous RMS gamma power PSD and the theoretical PSD for the ARX model.

### B. Aggregate Impact of Stimulation on RMS Gamma Power

For each of the 17 subjects, ten 4-second trials were collected using a sampling interval of 0.002 seconds. Henceforth, we will use actual time samples in seconds rather than integers to represent discrete time. Each trial can be represented as *x*_*k*_(*t*) where *t* = −2, −1.998, . . ., 1.998 are time samples corresponding to a sampling interval of Δ_*t*_ = 0.002 seconds and *k* = 1, . . ., 10. Stimulation was applied at *t* = 0. The ensemble average of the trials was then computed as

$$\bar{x}(t)=\frac{1}{10}\sum_{k=1}^{10}x_{k}(t)$$

**Fig. 5.**
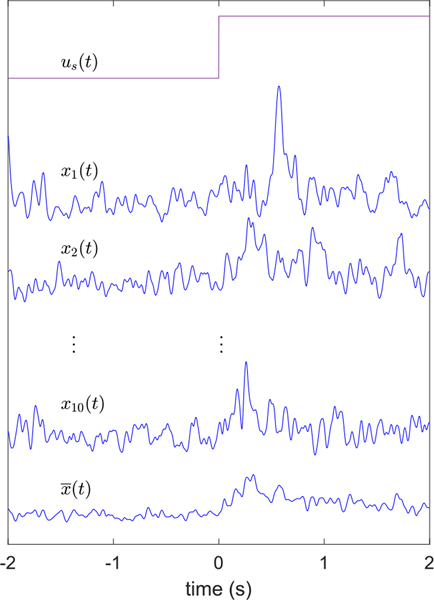
Instantaneous RMS gamma power trials, *x*_1_ (*t*), . . ., *x*_10_(*t*) and their ensemble average $\bar{x}(t)$. The stimulation signal amplitude *u*_*s*_(*t*) is a 2 mA step function.

**Fig. 6.**
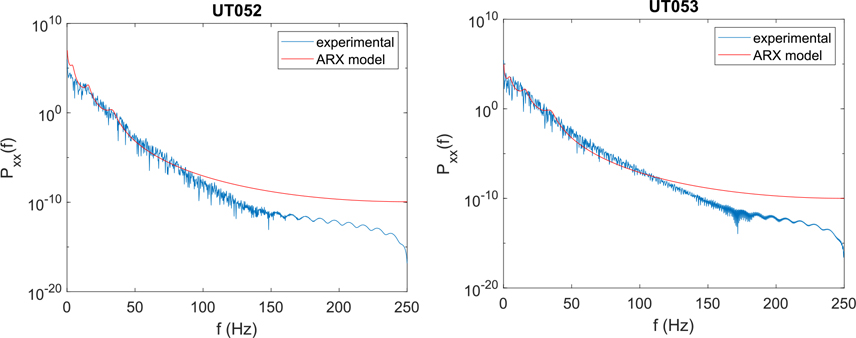
Comparison of averaged periodogram of instantaneous RMS gamma power (experimental) with theoretical power spectral density of identified ARX models, as given by (6) with *p* = 6.

**Fig. 7.**
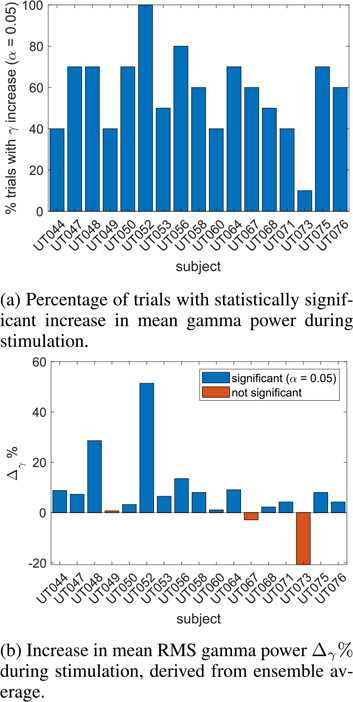
Impact of stimulation on mean gamma power.

An example of the data collected from a single subject is shown in Fig. 5. We compared RMS gamma power levels over a 2-second interval prior to stimulation onset with RMS gamma power over the 2-second interval immediately after stimulation onset. This reduced the possibility that long-term baseline drift in mean RMS gamma power levels affected our results. No-stimulation and stimulation RMS gamma power was compared two different ways. First, for each trial, we tested the null hypothesis that the mean of the no-stimulation data *x*_*k*_(*t*)*, t* = −2, . . ., −0.002 was equal to that of the stimulation data, *x*_*k*_(*t*)*, t* = 0, . . ., 1.998*, k* = 1, . . ., 10. A single-tailed t-test was used to test the null hypothesis for each of the 10 trials. The percentage of trials for which the null hypothesis was rejected at the 0.05 significance level (implying stimulation RMS gamma power levels are greater than no-stimulation levels) is shown in Fig. 7a. The second comparison was similar but rather than testing individual trials, we compared the mean of the no-stimulation ensemble average data, $\bar{x}(t)$, *t* = −2, . . ., −0.002 with the mean of the stimulation ensemble average data, $\bar{x}(t)$, *t* = 0, . . ., 1.998 using a single-tailed t-test. Fourteen of 17 subjects showed significant increases in mean RMS gamma power. Figure 7b shows the normalized increase in mean RMS gamma power levels,

$$\Delta_{\gamma }\%=\frac{{\bar{x}}_{s}\;-\;{\bar{x}}_{ns}}{{\bar{x}}_{ns}}\times 100\%$$

where

$${\bar{x}}_{ns}\equiv \frac{1}{1000}\sum_{t=-2}^{-0.002}\bar{x}(t),\;{\bar{x}}_{s}\equiv \frac{1}{1000}\sum_{t=0}^{1.998}\bar{x}(t)$$

In comparable no-stimulus experiments with the same subjects, only 8 of the 14 subjects showed significant RMS gamma power increases. The mean of Δ_*γ*_% was 7.8% during stimulation versus 2.8% during comparable no-stimulation trials, a significant difference (2-sided *t*-test, *p* = 0.05). This suggests that stimulation is likely to increase gamma power beyond the levels that would be expected from performing free-recall memory tasks alone.

### C. Controller Performance in Simulated System

**Fig. 8.**
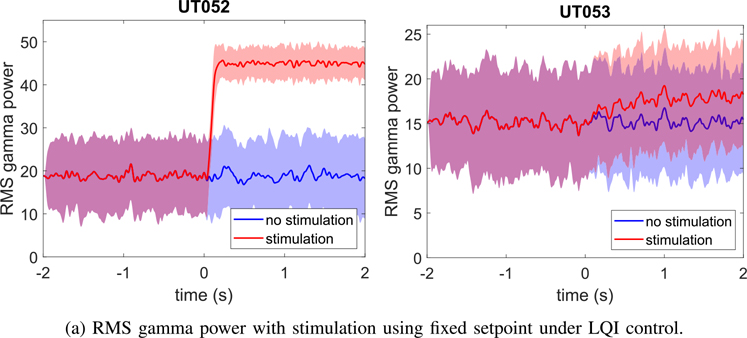
LQI controller simulations with control signal starting at *t* = 0 s for several subjects, showing mean and standard deviations over 100 independent trials.

**Fig. 9.**
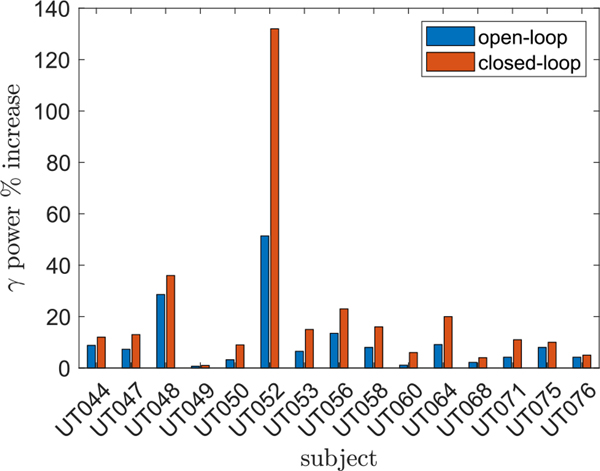
Percent increase in RMS gamma power (Δ_*γ*_%, see (17)) for open-loop (based on iEEG data) and closed loop conditions. The closed-loop results are based on simulated LQI control and had a mean of 22.8% across all subjects compared to 11.8% for the open loop condition.

**Fig. 10.**
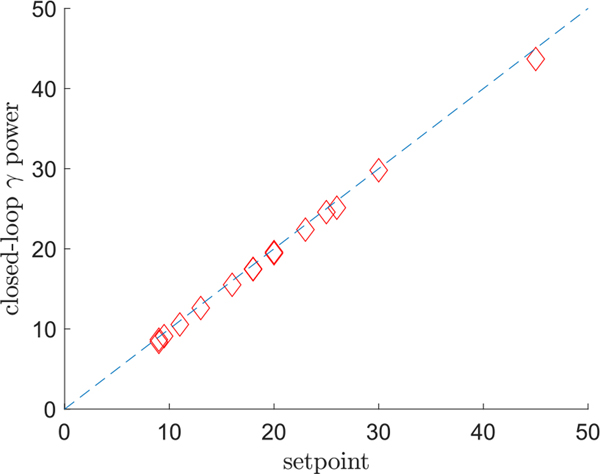
Simulated closed-loop RMS gamma power versus desired RMS gamma power setpoint.

We implemented an LQI servo-controller in Simulink using a sampling interval of 2 ms and a simulation time of 4 seconds ranging from *t* = −2 s to +2 s [35]. The parameters in the LQI cost function (15) were set to

(17) $$Q=\left[\begin{array}{cc} 0.005I_{6} & {\bar{0}}_{6}\\ {\bar{0}}_{6}^{T} & 100 \end{array}\right],\;R=1$$

where *I*_6_ is the 6 × 6 identity matrix and ${\bar{0}}_{6}$ is a 6 × 1 zero vector. The controller was started at *t* = 0 seconds. For each subject, the parameters for the LQI controller were dervied from the mean ARX parameters over all ten trials. The RMS gamma power setpoint was adjusted in order to keep the stimulation amplitudes under 9 mA, which, as discussed above, is within the safety limits for intracranial SEEG electrodes. Figure 8 shows the mean and standard deviation of RMS gamma power over 100 independent trials for two subjects. Figure 9 shows the normalized RMS gamma power increase for closed-loop and open loop-conditions for the 15 subjects who exhibited open-loop gamma power increases. The mean open-loop RMS gamma power increase for these subjects was 10.5%, as determined directly from iEEG, whereas for the closed loop LQI control simulations, the mean RMS gamma power increase was 20.8%. Figure 10 shows the simulated closed-loop RMS gamma power for each subject versus the desired setpoint. The normalized error was around −3% averaged over all subjects and was likely the result of stimulation being limited to 9 mA while the controller needed additional current to reach the setpoint.

## IV. Discussion

We created an LQI servo-control system developed from open-loop human brain stimulation data targeting the posterior cingulate cortex [8] with measured responses in the hippocampus. The larger goal of such a system is to improve memory performance in humans. The use of the PCC as a target region for neuromodulation rests in part on the ability to see predictable effects on RMS gamma power in the presence of stimulation, as suggested in Figure 7, as well as established anatomical connectivity in humans [36]. Certainly, it remains to be shown that a strategy targeting hippocampal gamma oscillations can improve memory performance across a large number of subjects. We intend to investigate the specific features of such as a system in subsequent experimentation. One option would be to identify narrow gamma frequency ranges that most strongly predict encoding success for an individual recording location, and then to model the impact of different stimulation frequencies on this signal. Such an approach would require varying the stimulation parameters used in system identification, as discussed below, but may represent a more efficient method for parameter identification as compared to the grid search approach used in existing closed loop systems for memory modulation [12]. We also note that we elected to focus on modulation of gamma rather than theta oscillatory activity. In rodents, restoration of pharmacologically or anatomically reduced theta activity is capable of restoring memory function [37]. However, human theta oscillations exhibit a greater diversity across a broad 2–10 Hz frequency range, and not all subjects exhibit persistent theta frequency power increases that predict successful encoding [9], [38], [39]. Targeting memory-relevant theta activity remains an active area of investigation; adjustment of PCC–applied stimulation parameters may be an effective approach given strong functional connectivity between the PCC and hippocampus during episodic memory processing [36], [40].

**Fig. 11.**
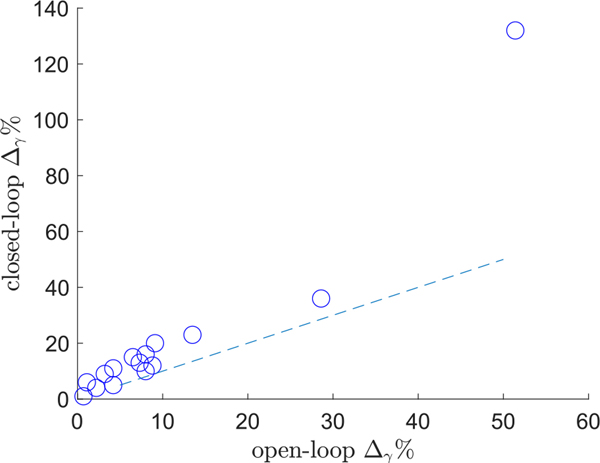
Percent RMS gamma power increases (Δ_*γ*_%, see Supplementary Material) for open-loop vs closed-loop stimulation. The dashed line has a slope of one. Larger open loop RMS gamma power increases predict larger increases under closed-loop stimulation.

We based our system identification parameters on brain stimulation data across 17 participants. In previous work, we established the safety of stimulation of the posterior cingulate cortex applied for a relatively long period of time (over 20 seconds), a distinct feature of our underlying data [8]. Moreover, focusing on hippocampal response to PCC stimulation permits relatively artifact-free recordings for modeling. Also, these underlying data are collected while individuals are *engaged in memory behavior*, which is a distinct advantage compared to approaches in which stimulation parameters are selected when the patients are at rest, or when stimulation is applied for a limited number of memory items [41]. However, our modeling suggests we can achieve reliable control of RMS gamma power at physiologically safe stimulus currents in in 15 of 17 subjects although some subjects only experienced modest increases in RMS gamma power with stimulation. The increase in mean RMS gamma power under closed loop control ultimately depends on the increase in mean RMS gamma power achievable under open-loop stimulation. This can be seen in Figure 11. The goal of the LQI controller is to arrive at some desired setpoint, as quickly as possible while minimizing the energy delivered to the patient. Open loop stimulation would require extensive trial and error to reach a desired gamma power setpoint. The closed loop approach makes it possible to reach a setpoint with considerably less effort. However our controller design cannot inherently generate higher gamma power than that which would be available via open loop stimulation.

In our proposed system, we decided to use an ARX model to characterize the effect of brain stimulation on neural activity. A linear model will not replicate the quadratically nonlinear nature of RMS gamma power, i.e., the model output can sometimes be negative, and the distribution of our model will be symmetric rather than skewed as would be expected from a quadratically nonlinear model. On the other hand, as detailed by Yang and others, linear models offer several advantages for design of a controlled system [18]. A significant disadvantage over nonlinear models is their complexity and computational burden, which hinders the ability to design powerful real-time closed-loop controllers. Using a linear ARX model, we are able to implement a robust state-space based neuromodulator while eliminating the need for a state estimator. These state-space based linear models have been successfully applied to complex dynamical brain systems for underlying surface EEG [42], magnetoencephalography [43], and local field potential data [44].

Improving the generalizability of our system will require that we establish its capabilities across a range of frequencies and that we understand how modulation of gamma power (for example) impacts other frequency ranges. Predictions of impact on non-gamma oscillations can be achieved with additional empirical data possibly combined with an improved plant model. More generally, regarding the goal of improving memory, the relative merits of a control system built on complex, multivariate brain signals versus a single (well-established) biomarker such as hippocampal gamma power remain a clear target of subsequent empirical investigation.

## V. Conclusion

The ability to achieve closed loop control of hippocampal gamma band power would impact the emerging field of neuromodulation to restore memory function. Our modeling data suggests that using the posterior cingulate cortex as a target for stimulation may be a propitious strategy. Our system identification schema utilizes previously obtained open-loop data to create a linear system model that describes the input-output relationship between stimulation to the PCC and neural activity in the hippocampus. Using an LQI servo controller designed based on our model, we were able to achieve control of hippocampal RMS gamma power, our biomarker for memory, in all patients on physiologically realistic time scales and at safe levels. We believe this strategy offers a promising approach for the neuromodulation of memory.

## VI. Supplementary Material

### A. Uniqueness of ARX Parameter Estimates for Least Squares Prediction Method

As stated in Section II-F, the parameter estimates are unique as long as the columns of the matrix *C* in (10), repeated here for convenience,

(18) $$C=\left[\begin{array}{ccccc} x(p) & \cdots & x(1) & u_{DC} & u_{s}(p+1)\\ x(p+1) & \cdots & x(2) & u_{DC} & u_{s}(p+2)\\ \vdots & \ddots & \vdots & \vdots & \vdots \\ x(N-1) & \cdots & x(N-p) & u_{DC} & u_{s}(N-p) \end{array}\right]$$

are linearly independent. This is a consequence of a well-known result on the solution of least squares systems of equations, which states that if the columns of a rectangular matrix *C* (having more rows than columns) are linearly independent, then the matrix *C*^*T*^ *C* is invertible (non-singular) and the least squares system of equations (9) will have a unique solution [29], [30]. It can be shown that the first *p* columns of *C* are linearly independent as long as the system function for the ARX model, given by

(19) $$\frac{1}{A(z)}=\frac{1}{1\;+\;\sum_{k=1}^{p}a_{k}z^{-1}}$$

is stable (roots of *A*(*z*) are inside the unit circle) [28]. Moreover, almost surely, the two right-most columns of *C* are linearly independent of the left-most *p* columns owing to the fact that one of the inputs to the model is the white noise signal *w*(*t*), which will make *x*(*t*) random, making it highly unlikely that these left-most *p* columns will be linearly dependent with the two deterministic exogenous input columns. Note that we refer to linear independence in the context of linear algebra (see for example [29]). It remains to show the extent to which the right two columns of *C* are linearly independent. For finite *N*, this is clearly the case since if *u*_*s*_(*t*) is a step function as defined in (3), there are no non-trivial linear combinations of these two columns that will produce a zero vector.

### B. Consistency of ARX Parameter Estimates for Least Squares Prediction Method

A parameter estimate is said to be consistent if the estimated parameters approach, almost surely, the actual values as the number of data points *N* used to form the estimate approaches infinity [28]. The consistency of the least-squares prediction method for estimating ARX parameters is examined in Section 7.1 of the textbook *System Identification* by by Söderström and Stoica (Prentice Hall, 1989), for the general ARX model [45]

(20) $$x(t)=-\sum_{k=1}^{p}a_{k}x(t-k)+\sum_{m=1}^{q}b_{m}u_{s}(t-m)+w(t)$$

The parameter estimates are consistent provided several conditions are met:

1. The matrix $\frac{1}{N}C^{T}C$ is non-singular as *N* → ∞.
2. The input signal *w*(*t*) is white noise.
3. The exogenous input *u*_*s*_(*t*) is persistently exciting of at least order *q*.

The latter condition insures that the matrix $\frac{1}{N}C^{T}C$ will be non-singular as the number of data points *N* approaches infinity. The order of persistence of excitation is related to the rank of the autocorrelation matrix of a signal [45]. For the case of a step function, it is shown in Example 5.3 of [45] that step functions are persistently exciting of order 1. Since in our model, *q* = 1, the parameter estimates we obtain are consistent. The Söderström and Stoica text doesn’t consider the DC exogenous input *u*_*DC*_ that we use, in addition to the step function *u*_*s*_(*t*). We now show that the matrix $\frac{1}{N}C^{T}C$ is non-singular only when *q* = 1 and that for *q* > 1, it becomes singular as *N* → ∞. Since we have already looked at the linear independence of the two right-most columns of *C* from the left-most *p* columns, we need only consider the linear independence of the two right-most columns of *C* as *N* → ∞. Define

(21) $$C_{2}=\left[\begin{array}{cc} u_{DC} & u_{s}(p+1)\\ u_{DC} & u_{s}(p+2)\\ \vdots & \vdots \\ u_{DC} & u_{s}(N-p) \end{array}\right]$$

Some straight-forward calculations show that with *u*_*s*_(*t*) (step function) and *u*_*DC*_ (constant) as defined in Section II-E

(22) $$\underset{N\to \infty }{\lim}\frac{1}{N}C_{2}^{T}C_{2}=\left[\begin{array}{ll} 1 & 1\\ 1 & 2 \end{array}\right]$$

which is non-singular as required for consistency. We note that if *q* = 2, if *u*_*s*_(*t*) is a step function, the parameter estimates will no longer be consistent. In this case we have

(23) $$C_{3}=\left[\begin{array}{ccc} u_{DC} & u_{s}(p+1) & u_{s}(p)\\ u_{DC} & u_{s}(p+2) & u_{s}(p+1)\\ \vdots & \vdots & \vdots \\ u_{DC} & u_{s}(N-p) & u_{s}(N-p-1) \end{array}\right]$$

which leads to

(24) $$\underset{N\to \infty }{\lim}\frac{1}{N}C_{3}^{T}C_{3}=\left[\begin{array}{lll} 1 & 1 & 1\\ 1 & 2 & 2\\ 1 & 2 & 2 \end{array}\right]$$

which is singular. Similar results are obtained for *q* > 2. Hence, a limitation of using a step function for *u*_*s*_(*t*) during system identification is that the ARX model cannot have *q* > 1 in (20), and still yield consistent estimates of the ARX parameters. This result is a consequence of step functions being persistently exciting of order 1. Rigorous proofs of these assertions are found in Complements C5.1, C6.1, and C6.2 of [45].

### C. Assessment of Model Quality Metrics

As described in Section III-B, 15 of the 17 subjects experienced RMS gamma power increases with stimulation, with 14 of these being significant at the *p* = 0.05 level. The ARX model parameters for our instantaneous RMS gamma power data were estimated using the Matlab function “arx”, which uses the linear least squares prediction solution to estimate the ARX parameters as described in Section II-F. The “arx” function returns a number of model quality metrics, including the mean squared prediction error $\left(\epsilon_{pred}^{*}\right)$ and the goodness of fit (FitPerc) given by (11) and (12), respectively. In order to assess these quality metrics we conducted a simulation where we identified the ARX paramters of an ARX random process with known parameters. For the ARX model to be identified, we chose the parameters: *a*_1_ = 5.6758, *a*_2_ = −13.6152, *a*_3_ = 17.6747, *a*_4_ = −13.0990, *a*_5_ = 5.2554, *a*_6_ = −0.8917, *b*_*DC*_ = 3.4689×10^−4^, *b*_*s*_ = 8.7828×10^−5^. These parameters, which were obtained from one of our subjects, were then used to generate a state-space model (see Section II-G). The state-space model was driven by a zero-mean Gaussian white noise sequence having a variance of 3.7197 × 10^−7^ as well as the exogenous inputs *u*_*s*_(*t*) (see (3)) and *u*_*DC*_(*t*) = 1*, t* = −2, . . ., 2. The output of the state-space model, *x*_*ARX*_(*t*), was then input to the “arx” function along with the known exogenous inputs. The identified parameters denoted by ${\hat{a}}_{k}$, *k* = 1, . . ., 6, ${\hat{b}}_{DC}$, ${\hat{b}}_{s}$ where compared with the actual parameters of the ARX model using the normalized squared estimation error,

(25) $$\frac{\sum_{k=1}^{6}{\left({\hat{a}}_{k}\;-\;a_{k}\right)}^{2}}{\sum_{k=1}^{6}a_{k}^{2}}$$

which was found to be 3.3972×10^−6^, while the corresponding error for the *b* parameters was 0.0209. The mean squared prediction error was given by $\epsilon_{pred}^{*}=3.8933\times 10^{-7}$ while the fit percentage (FitPerc) was 99.9943%. This was over a single run, but multiple runs yielded similar results. The fit percentage depends on the normalized mean squared prediction error. A small prediction error leads to a fit percentage close to 100 % (see Section II-F).

**Fig. 12.**
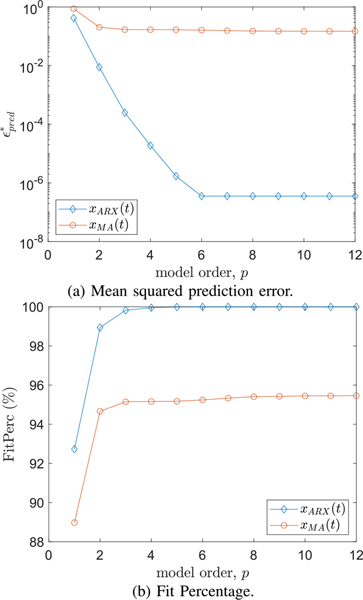
Model quality metrics for identifying the ARX parameters for two different signals: an ARX process of order 6, *x*_*ARX*_(*t*), and a moving average (MA) process of order 29, *x*_*MA*_(*t*) having similar spectral features.

Next we investigated the effects of under or overestimating the model order for the known ARX model. For model orders ranging from *p* = 1, . . ., 12, we computed $\epsilon_{pred}^{*}$, and FitPerc. To further evaluate these model quality metrics, we then computed the ARX model of a high-order moving average (MA) process given by

(26) $$x_{MA}(t)=\sum_{k=0}^{29}b_{k}w\left(t-k\Delta_{t}\right)+b_{DC}u_{DC}(t)+b_{s}u_{s}(t)$$

where *w*(*t*) is the same zero-mean Gaussian process used to produce *x*_*ARX*_(*t*). The *b*_*k*_ coefficients corresponded to a lowpass FIR filter (using the windowing method with a cutoff frequency of 1.25 Hz). These coefficients were scaled and the cutoff frequency was chosen so that the averaged periodogram of *x*_*MA*_(*t*) closely matched that of *x*_*ARX*_(*t*). The *b*_*DC*_ and *b*_*s*_ coefficients were chosen to yield the same DC values over the no-stim and stim intervals as *x*_*ARX*_(*t*). The results of both experiments are shown in Fig. 12. For the ARX process, the mean squared prediction error drops rapidly with increasing model order, and then levels off at the correct model order. Similarly the fit percentage rises rapidly as the model order is increased and then levels off at nearly 100% after the correct model order of *p* = 6. For the MA process the quality metrics deteriorated considerably compared to those for the ARX process, with no appreciable improvement in either of the quality metrics beyond an ARX model order of *p* = 3. These findings suggest that our approach to estimating ARX model parameters is accurate when the model being identified indeed corresponds to an ARX model, however attempting to identify ARX parameters of a signal that does not correspond to an ARX model (such as an MA model) will have a negative impact on model quality metrics.
